# Observations on the perfusion recovery of regenerative angiogenesis in an ischemic limb model under hyperoxia

**DOI:** 10.14814/phy2.13736

**Published:** 2018-06-21

**Authors:** Luis Monteiro Rodrigues, Henrique Silva, Hugo Ferreira, Marie‐Ange Renault, Alain‐Pierre Gadeau

**Affiliations:** ^1^ CBIOS — Universidade Lusófona's Research Center for Biosciences and Health Technologies Lisboa Portugal; ^2^ Pharmacology Science Department Faculty of Pharmacy Universidade de Lisboa Lisboa Portugal; ^3^ IBEB — Biophysics and Biomedical Engineering Institute Universidade de Lisboa Faculty of Sciences Lisboa Portugal; ^4^ Biology of Cardiovascular Diseases Universite Bordeaux Montaigne Inserm Pessac France

**Keywords:** Hind limb ischemia, hyperoxia, laser Doppler, mice, perfusion balance between limbs

## Abstract

This study combines two well‐known vascular research models, hyperoxia and hind limb ischemia, aiming to better characterize capacities of the hyperoxia challenge. We studied two groups of C57/BL6 male mice, a control (C) and a hind limb ischemia (HLI) group. Perfusion from both limbs was recorded in all animals by laser Doppler techniques under an oxygen (O_2_) saturated atmosphere, once for control and, during 35 days for the HLI group. We used a third set of normoxic animals for HLI morphometric control. The expected variability of responses was higher for the younger animals. In the HLI group, capillary density normalized at Day 21 as expected, but not microcirculatory physiology. In the operated limb, perfusion decreased dramatically following surgery (Day 4), as a slight reduction in the non‐operated limb was also noted. Consistently, the response to hyperoxia was an increased perfusion in the ischemic limb and decreased perfusion in the contralateral limb. Only at Day 35, both limbs exhibited similar flows, although noticeably lower than Day 0. These observations help to understand some of the functional variability attributed to the hyperoxia model, by showing (i) differences in the circulation of the limb pairs to readjust a new perfusion set‐point even after ischemia, an original finding implying that (ii) data from both limbs should be recorded when performing distal measurements in vivo. Our data demonstrate that the new vessels following HLI are not functionally normal, and this also affects the non‐operated limb. These findings confirm the discriminative capacities of the hyperoxia challenge and suggest its potential utility to study other pathologies with vascular impact.

## Introduction

In vivo models have been extensively used to approach mechanisms involved in microcirculatory pathophysiology (Couffinhal et al. 1998; Monnet and Teboul 2008; Silva et al. 2015a, 2017). All models, human or animal, have limitations, primarily because no single model is completely capable of representing one disease or regulatory mechanism (Strain et al. 2012; Silva et al. 2017). Moreover, most of the models are far from fully characterized or normalized, making a comparison of results an impossible task (Silva et al. 2015a).

Oxygen (O2) homeostasis is a principal issue in research, and is used as a stressor in many (human) circulatory models (Nyberg et al. 2015) due to its powerful vasoactive properties. While exposure to hypoxia led to the identification of several physiological responses, including the discovery of hypoxia‐inducible factor 1 (HIF‐1) (Semenza and Wang 1992), central and peripheral mechanisms involved in hyperoxaemia have not been completely identified.

The direct effect of hyperoxia and hyperoxemia reported by many studies is vasoconstriction (Rousseau et al. 2005; Bak et al. 2007; Sinski et al. 2014), but contradictions still arise. Extraordinary heterogeneity among studies, experimental protocols, and quantification instruments are certainly contributors to this conflict, from the definition of hyperoxemia to the exposure periods, as well as the time of assessments during hyperoxia (Bak et al. 2007; Sinski et al. 2014; Silva et al. 2015a). Nevertheless, it is widely regarded as safe to apply in human studies and is frequently used in vascular research. Hyperoxia seems to deactivate carotid body chemoreception, reducing sympathetic activity, evoking a negative feedback between chemoreflex and baroreflex, such that inhibition of the carotid body triggers the baroreflex response (Despas et al. 2012; Sinski et al. 2014). Locally, oxygen inhibits NO release such that vasoconstriction is the expected response in most vascular beds. However, a perfusion increase has also been reported (Bongard et al. 1992; Silva et al. 2016) with no convincing explanation.

We developed an experimental model in mice combining hyperoxia, measurements in both limbs with two laser Doppler systems, and surgically‐induced Hind Limb Ischemia (HLI). HLI is one of the most popular models used to explore many aspects of the in vivo vascular biology and is also used to study peripheral artery disease and chronic severe limb ischemia (Couffinhal et al. 1998; Paek et al. 2002; Zapata et al. 2009; Hellingman et al. 2010). It was not our goal to look for therapeutic benefits of hyperoxia on vascular growth and limb reperfusion or to evaluate the impact of hyperoxia on HLI reperfusion and angiogenesis. Rather, our goal is to better identify and understand some of the difficulties and controversies involving hyperoxia that limit its use. All perfusion changes from hind limbs (both the operated and contralateral) were recorded with a combination of real‐time laser Doppler flow (LDF) and imaging (LDI) to minimize eventual experimental bias resulting from using only a contact method (LDF), strengthening the related quantitative analysis. Our primary objective was to develop a reproducible, normalized animal model, capable to detect any early potential functional impacts to microcirculation, from different pathologies.

## Materials and Methods

### Animals

Twenty‐five male C57/BL6 mice were assigned to the hyperoxia challenge experiment. The control (C or simply “control”) group included animals with different ages (a younger set A, *n* = 8 weeks old (w.o.), 22.7 ± 1.0 g, an intermediate set B, *n* = 9, 16 w.o. 24.2 ± 2.2 g, and an older set C, *n* = 8, 28 w.o., 28.5 ± 5.9 g). The subgroup with 16 w.o. was later submitted to the hind limb ischemia (HLI) procedure. Another group with 48 male C57/BL6 mice (14 ± 2 w.o.) was used for morphometric analysis after HLI. Animals were installed at the INSERM U1034 animal facility, with controlled temperature and humidity conditions (21 ± 1°C, 40–60%), exposed to regular 12 h light/12 h darkness cycles with food and water ad libitum. Animal experiments were performed in accordance with the guidelines from Directive 2010/63/EU of the European Parliament on the protection of animals used for scientific purposes, approved by the local Animal Care and Use Committee of Bordeaux University, complying with recently published principles and standards for reporting animal experiments (Grundy 2015).

### Experimental

All procedures were conducted under controlled temperature (21 ± 1°C) and humidity (40–60%).

#### Anesthesia

For data collection, animals were anesthetized by an intraperitoneal administration of a saline mixture of ketamine (125 mg/kg, Imalgene, Merial, USA) and xylazine (10 mg/kg, Ronpum, Bayer, Germany), which provided a sedation period of 50 min. Animals submitted to the HLI procedure were anesthetized with 5% isoflurane (AErrane, Baxter SAS, USA) in 100% oxygen, reduced to 3% during surgery.

#### Hyperoxia challenge procedure

Under anesthesia, animals were laid horizontally onto a surgical pad placed on top of an electric mat (Rainforest Heat Wave Mat Substrate Heater, Exo‐Terra, USA) kept at 36°C. Their heads were secured to the pad by a proper adapter, while the respective hind limbs were lateralized. Two LDF probes were attached by double‐sided adhesive strips (PF 105‐3 Double‐sided Tape Strips, Perimed, Sweden) to the inferior aspect of both paws, one probe for each paw, near the posterior limit of plantar footpads. LDF recordings were taken in three phases, each of 10 min duration—a stabilization phase (Phase I), with animals breathing the room atmosphere; a challenge phase (Phase II), with animals breathing a saturated oxygen atmosphere (inhalation fraction ≅ 100%); and a recovery phase (Phase III), after returning to room atmosphere. As discussed ahead, exposure time to oxygen seems to a critical determinant of the responses following. These timings were chosen based on our experience on human exposure to hyperoxia, as a similar response pattern was found in mice (Zapata et al. 2009; Silva et al. 2015a,b, 2016). The oxygen content was ensured by a suitable cylinder connected to the pad (AirLiquide, France). LDI measurements were also obtained in the HLI group at the end of the recovery period.

#### Hind limb ischemia procedure

HLI procedure was induced in the left limb of nine C57BL/6 mice (subgroup B) by ligation and removal of the superficial femoral artery and vein as previously published (Nyberg et al. 2015). Thirty minutes before anesthesia and surgery, analgesia was induced in all animals by injection of 30 *μ*g/kg Buprenorphin (Mallinckrodt Pharmaceuticals Mulhuddart, Ireland). A longitudinal incision was made from the knee to the medial region of the hip and the superficial adipose tissue removed to expose the superficial femoral artery and vein. These vessels, together with the femoral nerve, were then ligated proximally at the level of peritoneum and distally at the level of the knee. Vessels were then excised throughout their entire course, and the incision closed with PROLENE 7‐0 polypropylene surgical wire (Ethicon, USA). Postoperative analgesia was maintained by a second Buprenorphin injection (30 *μ*g/kg) 8 h after surgery. At the conclusion of the study segments, mice were euthanized.

**Figure 1:**
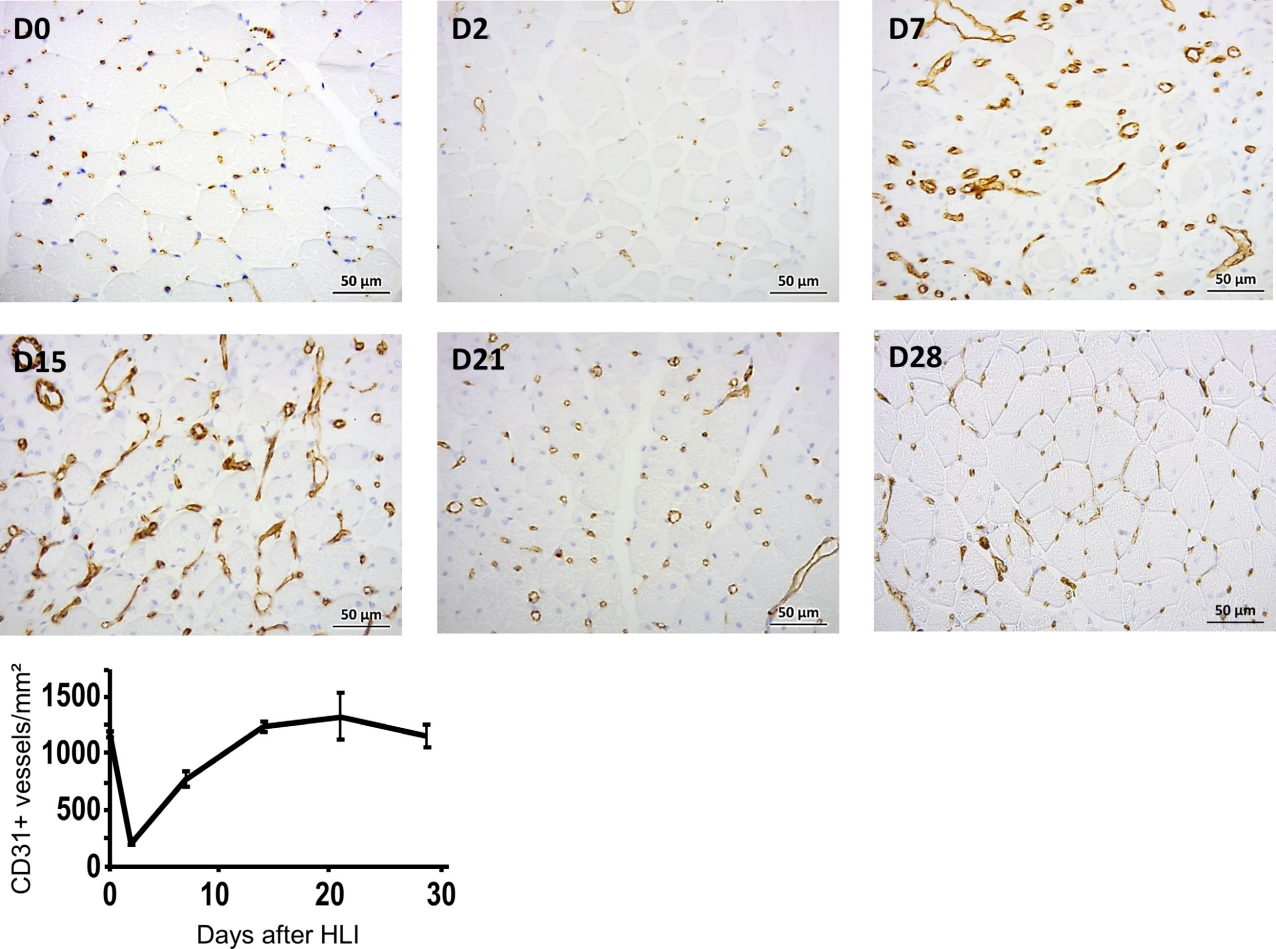
Representative images of ischemic muscle obtained from Day 0 until Day 28 in a group of normoxic animals submitted to HLI. Capillary density was evaluated after CD31 staining of ischemic tibialis anterior muscle sections. The graph represents mean vessels/mm² and the error bars represent variability between mice (*n* = 6) per time.

In the morphometric analysis group, the tibialis anterior muscle was harvested at days 0, 2, 9, 15, 21, 28, and fixed with methanol before paraffin inclusion. These animals were not submitted to the hyperoxia challenge, rather, the group was included to evaluate HLI‐related morphometrics. The capillary density was evaluated after CD31 staining (rat anti CD31, BD Pharmigen) of normal and ischemic muscle sections and quantified by counting CD31+ vessels per mm² on the entire section. Representative pictures were obtained under a Zeiss AXIO Scope A1 microscope using a × 40 objective and captured with an Axiocam 105 digital color camera. Vessels were counted in 10 microscope fields from six mice per group (Fig. 1).

#### Blood perfusion data collection

The hyperoxia challenge procedure was applied to both control (C) and HLI groups on Day 0, before surgery, and repeated on the HLI group in Days 4, 6, 9, 12, 15, 21, and 35 of the experimentation period.

Blood perfusion was primarily quantified by LDF, expressed in arbitrary blood perfusion units (AU). LDF signals were collected at a sampling frequency of 32 Hz, using a time constant of 0.01 sec, from two PF 407 probes connected to two PF5010 modules of a Periflux 5000 system (Perimed, Sweden). The vascular response to the challenge was quantified by the perfusion changes in each phase, expressed in absolute values (arbitrary blood perfusion units, AU). The microcirculation recovery profile was evaluated by the control‐to‐ischemia resting flow difference and by the ratio of ischemic‐to‐control resting blood flow, both obtained from the final 5 min of Phase I LDF recording values.

Perfusion imaging was obtained by the MoorLDI2‐IR Laser Doppler Imager (Moor Instruments, UK) in Days 4, 6, 9, 12, 15, 21, and 35, after the end of stabilizing period (Phase III) of the hyperoxia phase procedure. Scans were obtained in a shaded room with constant light, temperature (22 ± 1°C) and humidity (40–60%) conditions, at a speed of 10 msec/pixel and with a 31 cm distance between the scan head and the board. The distance between the scan head and the animal was kept constant in all measurements. Regions of interest (ROI) were manually identified from the paw up to mid‐leg, and blood flow expressed as the chromatic intensity in arbitrary perfusion units (AU). The ratio of ischemic‐to‐control blood flow was calculated to avoid the influence of room light on LDI blood perfusion readings.

### Statistics

Data analysis was focused on the last 5 min of Phases I and II, and the entire 10 min of Phase III. These timings were chosen based on our previous experience in the application of this method to humans (Silva et al. 2015a) while also considering the need to avoid instability related to adaptation to and cessation of anesthesia.

LDF means were compared by the Wilcoxon signed‐rank test and, between paws, by the Mann–Whitney *U* test for independent samples. LDF and LDI absolute values were compared by linear correlation, while ischemic‐to‐control ratios and control‐to‐ischemia differences were tested by the Spearman's Rho coefficient. An intraclass correlation *F* test using average measures was applied to assess the reliability of LDF and LDI results. A significance level of 0.05 was adopted.

## Results

### Control group

At Day 0, all animals presented similar hind limb circulatory conditions, regardless of age. As shown (Table 1), no significant differences could be found regarding baseline perfusion conditions in both limbs for any of the subgroups (8, 16, and 28 weeks old). The same comment is applicable for the recovery periods. The hyperoxia challenge only revealed significant differences between the right and the left limbs in the youngest animals (A, *P *= 0.012) and between the right limbs of subgroups A and B (*P *= 0.009). This variability of response is similar to what is described in human (Zapata et al. 2009; Sinski et al. 2014; Silva et al. 2015a) with a recurrent response (50%) of decrease of blood perfusion. Our protocol included recordings from both limbs, not a typical experimental procedure, as will be discussed later in the text, which allowed us to detect different responses between the two limbs responding to hyperoxia in the non‐operated group (Table 1, individual results not shown). These included a simultaneous perfusion decrease in both limbs (PerD), a simultaneous perfusion increase in both limbs (PerI), and a third type of response which we termed a mixed response (PerMix). In this third response, not previously described, a perfusion decrease was observed in one limb while the contralateral limb showed a perfusion increase. In summary, we found:

☐ In subgroup A (8 w.o.) a simultaneous perfusion decrease in both limbs (PerD) in five mice, while three exhibited a mixed response;
☐ In subgroup B (16 w.o.) a perfusion decrease in both limbs (PerD) in two mice, four exhibited a mixed response, and three others a perfusion increase in both limbs (PerI)
☐ in subgroup C (28 w.o.) a perfusion decrease in both limbs (PerD) was registered in one mouse, five exhibited a mixed response, and two others a perfusion increase in both limbs (PerI).

**Table 1:** Blood perfusion (BP) measured by LDF (AU's, mean + *sd ‐ standard deviation*) in animals included in the control (n=25) group. Only animals from subgroups A and B were submitted to the O_2_ challenge procedure; subgroup C animals were only used for morphometric follow‐up of the HLI procedure

Control group	Blood flow (AU)	Baseline	Challenge	Recovery	Variability (%) change (baseline to challenge)
Subgroup A (8 weeks old, *N* = 8)	Right limb	137.0 ± 22.8	118.8 ± 23.3	144.5 ± 17.5	−13.2
	*P* value	–	0.012*	0.123	–
	Left limb	131.6 ± 22.7	115.4 ± 32.8	132.7 ± 35.2	−13.2
	*P* value	–	0.091	0.611	–
Subgroup B (16 weeks old, *N* = 9)	Right limb	159.3 ± 34.5	163.7 ± 31.5	171.9 ± 49.8	5.5
	*P* value	–	0.499	0.310	–
	Left limb	173.1 ± 46.4	161.1 ± 45.5	184.1 ± 56.5	−7.1
	*P* value	–	0.161	0.069	
Subgroup C (28 weeks old, *N* = 8)	Right limb	159.2 ± 37.1	169.9 ± 53.6	200.0 ± 73.3	5.6
	*P* value	–	0.263	0.036*	–
	Left limb	160.5 ± 58.2	166.5 ± 59.6	180.8 ± 68.8	5.0
	*P* value	–	0.575	0.123	–
*P* value (A vs. B, right limb)		0.094	0.009*	0.152	–
*P* value (A vs. B, left limb)		0.210	0.072	0.072	
*P* value (A vs. C, right limb)		0.195	0.105	0.382	
*P* value (A vs. C, left limb)		0.336	0.054	0.072	
*P* value (B vs. C, right limb)		0.779	0.867	0.867	
*P* value (B vs. C, left limb)		0.721	0.721	0.959	

Statistical comparison of means from both paws in each subgroup and between subgroups, before surgery and in the subsequent experimental phases, showed no relevant differences between limbs or between subgroups (*P < 0.05; ns, no significance).

A simple analysis of variability comparing the responses to the challenge within each subgroup (Table 1) revealed that the highest variability (−13.2%) was noted in the youngest subgroup, where the decrease in perfusion was the predominant response (equivalent in both paws). This variability explains the differences referred to above, only detected with the youngest group as it seems to decrease and even reverse with increasing age (Table 1)

### HLI group

**Figure 2:**
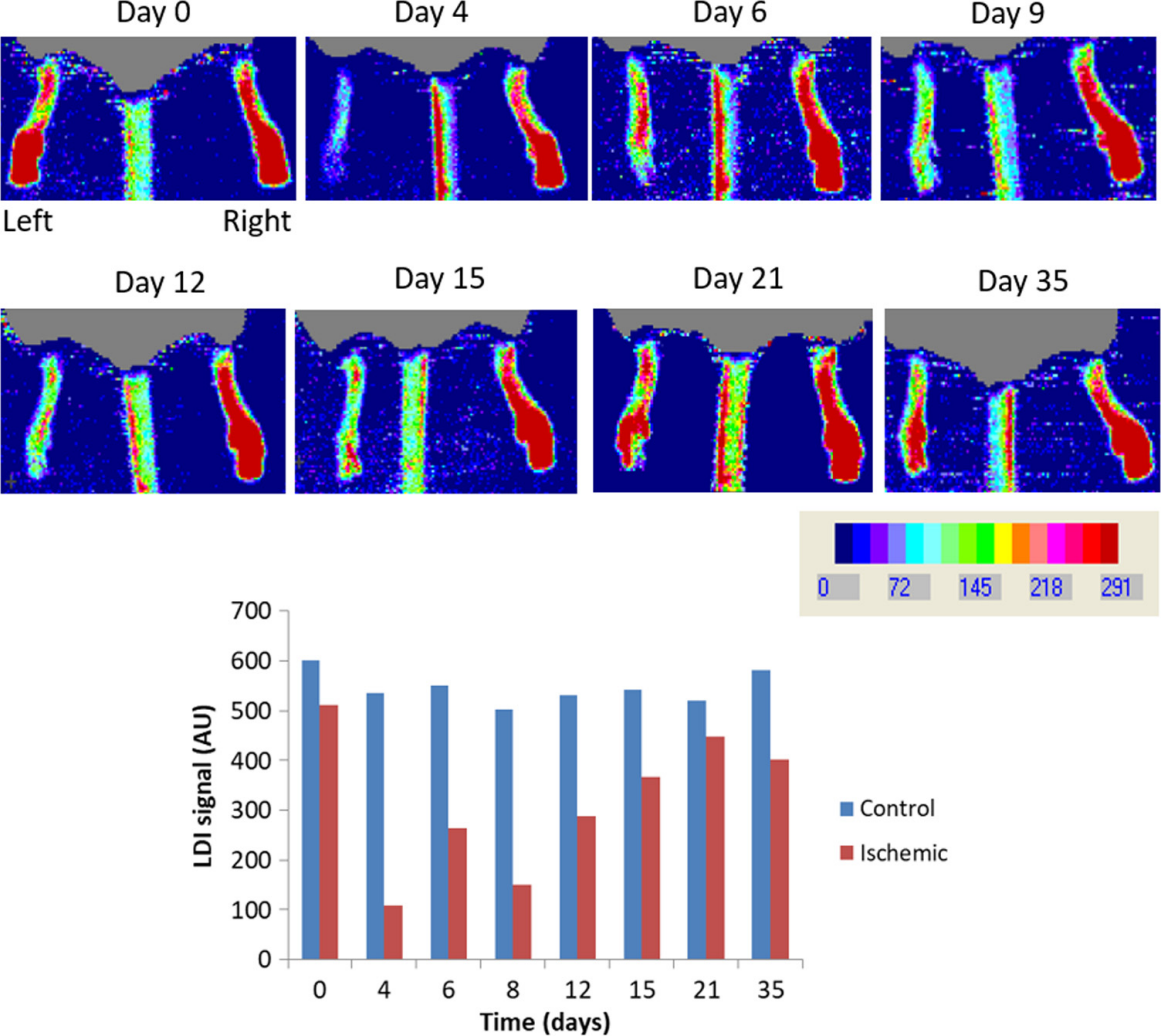
An illustrative example of the blood perfusion recovery registered by LDI in one animal from the HLI group (*n* = 9) following the HLI procedure. Top–Control (right) and ischemic (left) limb perfusions were registered on Days 0 (before surgery), 4, 6, 9, 12, 15, 21, and 35. Bottom–Blood perfusion (in AU's) was calculated as a function of the chromatic intensity is shown in the graph to illustrate the particular perfusion changes detected in both limbs after ischemia (see text).

In the HLI group, all animals recovered well from the surgery, showing a progressive and expected use of the injured limb from the first week onward. Feet perfusion measured by LDI also showed the same progressive recovery (Fig. 2). The quantification of perfusion changes with two laser Doppler techniques (LDF and LDI) was implemented to reduce eventual experimental bias. Although both are based on the same principle, a non‐contact imaging technique could help reduce potential errors and facilitate our rationale. Both laser Doppler techniques provided coherent, reproducible results and equally useful parameters for the perfusion quantification follow up and recovery.

**Figure 3:**
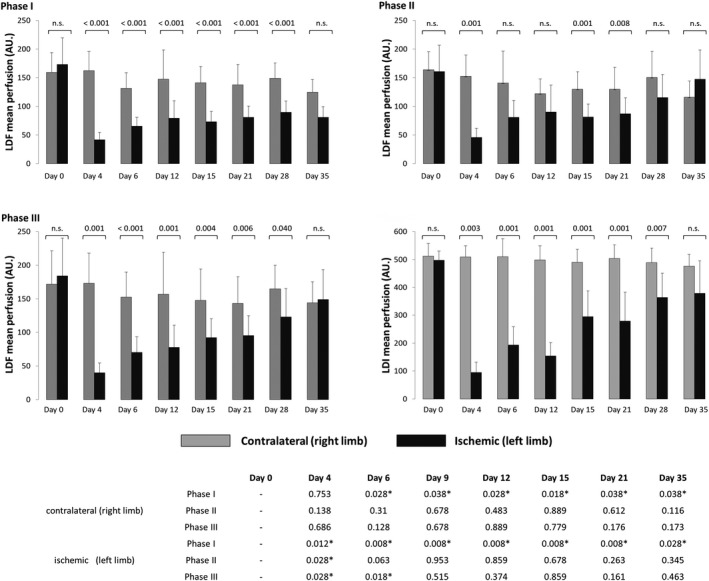
Graphical representation of the blood perfusion data (mean + SD) obtained throughout the experimental period in the HLI group (*n* = 9). LDF measurements took place in each phase of the O2 procedure, while LDI measurements were obtained after the stabilization phase (Phase III) of the O2 challenge (see text). Comparative significance of perfusion changes between limbs is shown over each bar graph pair, and for each limb during the experimental period relative to Day 0 in the lower table (see text) (*P < 0.05).

Figure 3 shows the evolution of blood perfusion registered by LDF and LDI on control and ischemic limbs throughout the experimentation period. The first measurement after surgery took place on Day 4, and a significant decrease of the baseline (Phase 1) perfusion on the ischemic limb was immediately detected, compared with Day 0 (*P* < 0.012). This difference from Day 0 was significantly maintained in the subsequent measurement days until Day 35, the end of the experiment (*P *= 0.028). Nevertheless, the ischemic limb perfusion recovery was visible throughout the evaluation period (Fig. 3). Regarding the contralateral limb, a surprising, progressive reduction of perfusion compared with Day 0 was also detected in Phase 1, significant from Day 6 until the end of the experiment. During the recovery period, the ischemic limb perfusion continued to increase until Day 35, while the control limb showed lower values, relative to Day 0, in all phases of the experiment. Comparing the perfusion performance of both limbs during the experiment, we found significant differences (*P* < 0.001) between both limbs in Phase I for each recorded day from Day 6 to Day 21. By Day 35, differences between the paired limbs could no longer be detected, signaling full recovery and the end of the experiment (Fig. 3).

**Figure 4:**
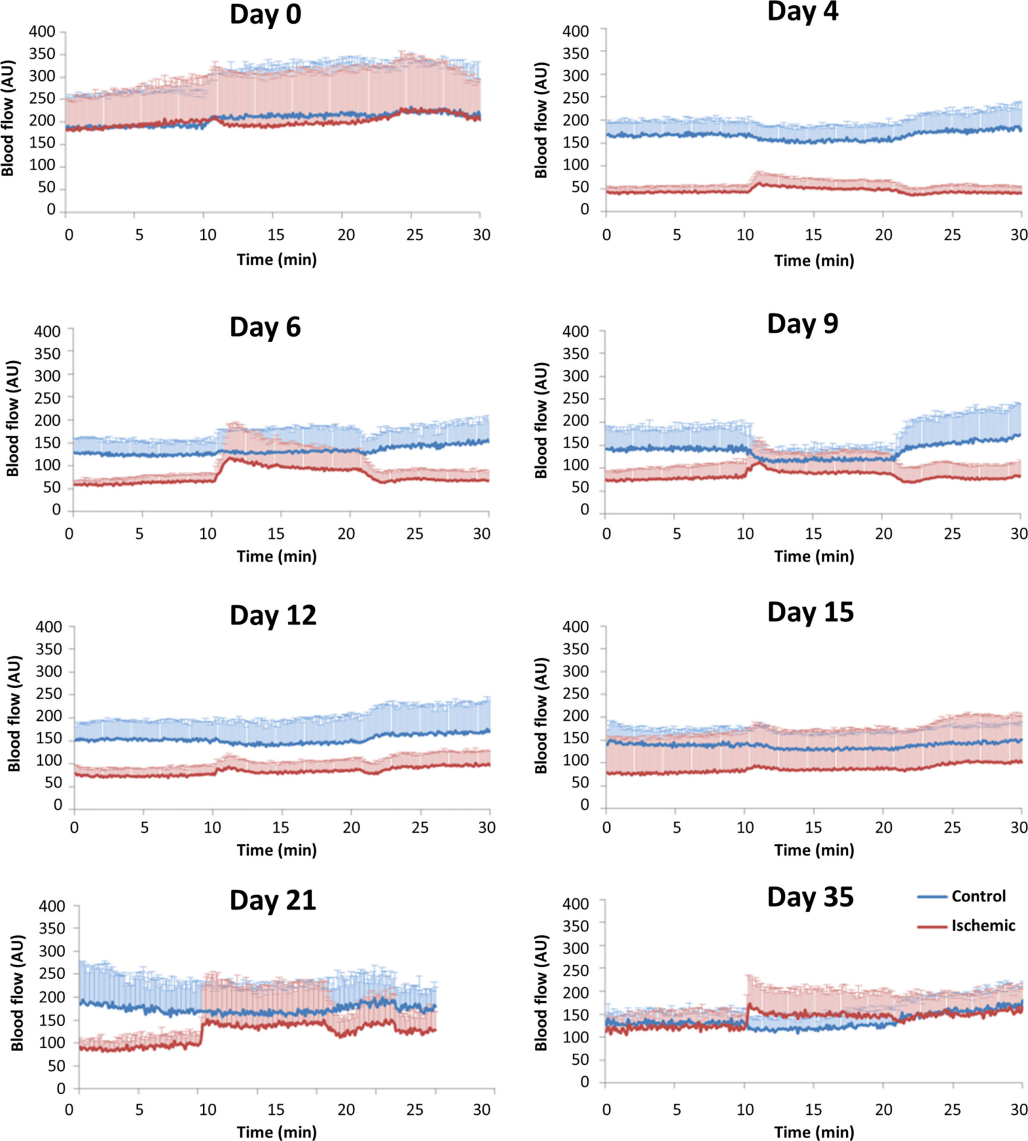
Graphic representation of both hind limbs perfusion variations (HLI group, all animals *n* = 9) measured by LDF and registered during the complete experimental procedure (all phases) after HLI. The ischemic (red) and the contralateral (blue) limb flows are mean values (standard deviations are not shown to provide a clearer view of the perfusion evolution). A new perfusion flow steady state is reached by Day 35, lower than Day 0, showing no significant differences between limbs (see text).

Comparing the hyperoxia challenge and the recovery phase responses in each limb of HLI group with Day 0 (Fig. 3), we found that:

☐ In the ischemic limb, the response to challenge was always significantly different from Days 4 to 35, while no significant differences were observed from Day 9 onward regarding the recovery after hyperoxia.
☐ In the contralateral limb, the challenge and the recovery responses from Day 4 to 35 were not significantly different from Day 0 (Fig. 3)
☐ The hyperoxia challenge (Phase II) consistently induced a reduction of perfusion in the non‐operated limb from Day 4 onward, while the ischemic limb always responded with a perfusion increase (Fig. 4). This behaviour was confirmed in all the experiments and will be further discussed later in the text.

LDI measurements were only obtained after the stabilization period following the hyperoxia procedure. The Day 4 measurement on the ischemic limb indicated a significant perfusion decrease (*P *= 0.012), followed by a progressive increase toward Day 35, consistently lower than Day 0 (Fig. 3). On Day 35, however, no significant differences were observed, indicating full perfusion recovery. No relevant changes were detected during this period for the contralateral limb perfusion, but a consistent, statistically nonsignificant, reduction of flow in the right limb was also present (Fig. 3). Comparing the perfusion in both limbs during this period, we observed statistically significant differences (*P* < 0.05) between both limbs for every day in all phases from Day 6 to Day 21. As indicated previously, by Day 35 perfusion differences between both limbs could not be detected (*P *= 0.085). As with LDF, this confirmed the normalization of the tissue's perfusion condition (Fig. 3).

**Figure 5:**
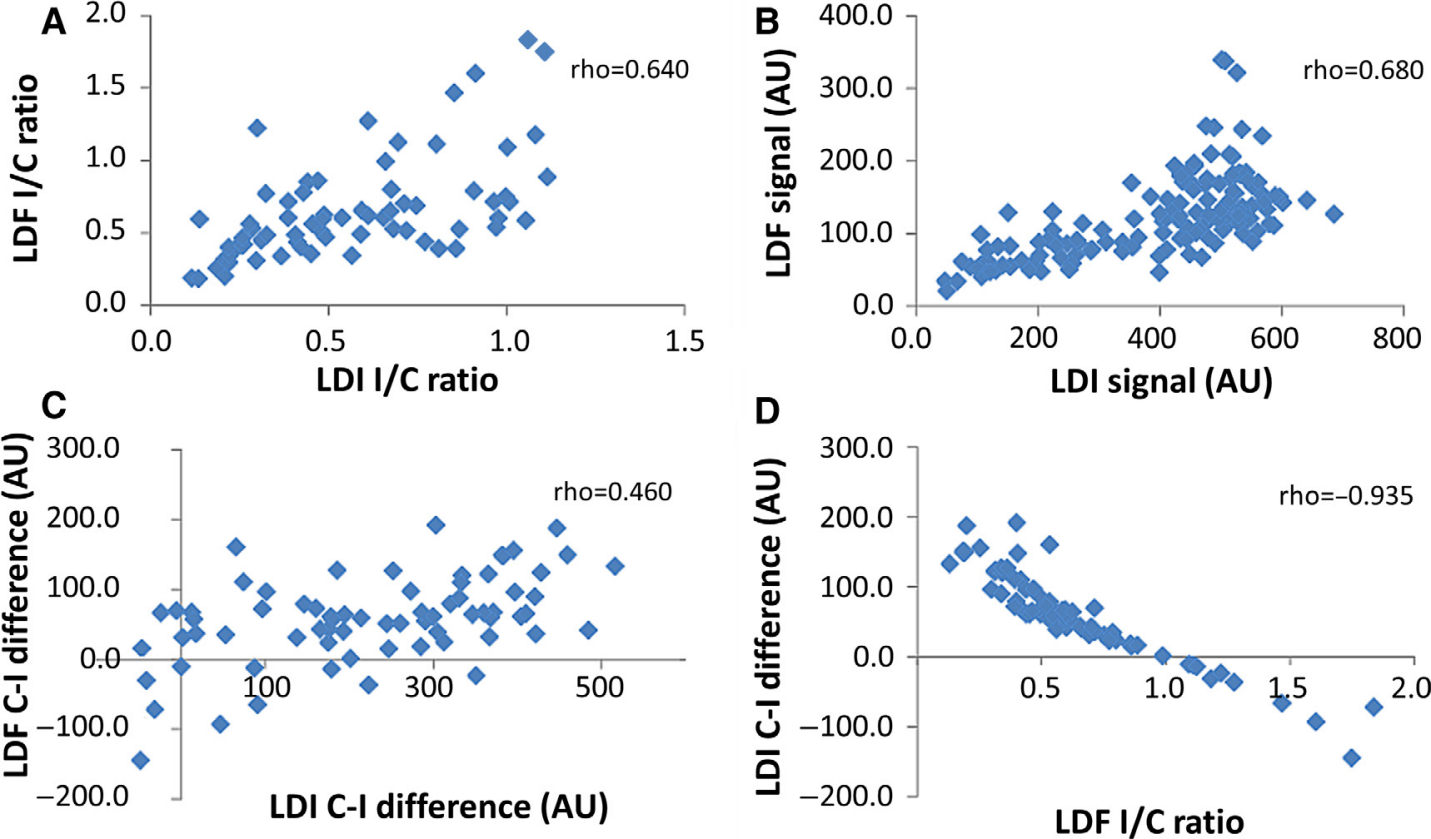
Correlations between LDI and LDF quantifiers obtained in the HLI group (*n* = 9). (A) LDI and LDF ischemic‐to‐control ratio, (B) LDI and LDF control‐ischemic difference, (C) LDF ischemic‐to‐control ratio, and (D) LDF control‐ischemic difference.

Spearman and intraclass correlations between measurement parameters revealed significant positive correlations between mean blood perfusion (*P* < 0.001), the ischemic‐to‐control ratio (*P* < 0.001) and control‐ischemic difference (*P* < 0.001), obtained by LDF and LDI, and a significant negative correlation between ischemic‐to‐control ratio and control‐ischemic difference (*P* < 0.001) (Fig. 5). The intraclass *F* correlation test revealed a significant correlation between mean blood perfusion (*P* < 0.001), the ischemic‐to‐control ratio (*P* < 0.001) and control‐ischemic difference (*P* < 0.001), obtained by LDF and LDI. Furthermore (Fig. 5), these correlations demonstrated that the detected changes are consistent and the quantifiers used from either technique are appropriate to analyze microcirculatory behavior in this type of study.

## Discussion

The use of hyperoxia as a physiological challenge to explore in vivo microcirculatory function has been limited due to a lack of experimental standards and procedures (Dantzker et al. 1974; Abman 1994; Jaffal et al. 2017) but also to a poor knowledge about the mechanisms to explain the wide variability of related responses (Abman 1994; Potter et al. 1999; Jaffal et al. 2017). Our search for a better use of this powerful instrument (O2) was primarily made using a standardized challenge procedure involving a 10 min hyperoxia challenge followed by a 10 min normoxic recovery, in which all perfusion changes were recorded in both distal limbs. Furthermore, by associating this procedure to the well‐known HLI model, we could test the relevance of the gathered information and, eventually, have a different functional view into the model.

In our experimental conditions, we confirmed that the primary physiological response to hyperoxia and hyperoxemia is the reduction of perfusion (Rousseau et al. 2005; Bak et al. 2007; Sinski et al. 2014), but also detected that the response measured by LDF could differ between the two limbs, a finding (to our knowledge) not previously described. Moreover, we noticed that the mixed perfusion events were more frequent in the older subgroups, where variability of response seems to be more consistently reduced (Table 1). We have considered that the reported variability may be related to the variability of the LDF recordings, due to its pulsatile (difficult to handle) nature. However, we also speculate that these mixed responses may result from a circulatory homeostatic adaptation between both limbs, likely centrally mediated. We will need more data to confirm these views, and keep in mind that animals are anesthetized with a mixture known to modulate cardiovascular activity, far from the normal physiological state.

When applied to the HLI group, our model also suggests a perfusion balance between the operated and non‐operated limbs from Day 4 until Day 35. At Day 4, before the oxygen challenge, a pronounced perfusion reduction in the ischemic limb is already obvious. From Day 4 onward, hyperoxia consistently evokes a (slow) perfusion increase in this limb until the end of the study (Day 35), where values are lower than Day 0 (Fig. 4). Meanwhile, in the contralateral limb, perfusion also slightly decreased before the oxygen challenge relative to Day 0. In other words, ischemia immediately reduced perfusion in the affected limb, but it also impacted the contralateral limb where a reduction of perfusion is detected from Day 4 on, and this reduction is statistically significant from Day 6 onward. In this limb, hyperoxia consistently evokes a perfusion decrease (Fig. 4) until by Day 35, at which time these perfusion differences between limbs are no longer seen (Figs. 3 and 4). The morphometric analysis of the capillary density, from normoxic animals (Fig. 1) confirmed a significant increase in capillary density between Days 21 and 35, in which the blood perfusion progression reaches a plateau between 21 and 28 days after the procedure (Couffinhal et al. 1998; Paek et al. 2002; Renault et al. 2013). However, our functional approach shows a different but consistent finding, unrelated in our opinion, with the direct effect of hyperoxia, only sporadically applied (for 10 min in the measuring days). Rather, it unambiguously demonstrates that, following ischemia, the vascular physiology of the new vessels is not normal and that fact affects the limb pair as a unit. The microcirculatory physiology critically depends on a highly structured network of vessels arranged to optimize oxygen delivery to the tissues. In turn, oxygen is also a critical regulatory element of these functions, not only acting locally, influencing resistance, but also controlled by other local and systemic (homeostatic) regulators. The capacity to recover muscle vascularization, as occurs with the HLI procedure, may not be equated with a full functional recovery of the muscle, especially in the presence of this regional (limb pair) balance. A recently published paper (Arpino et al. 2017) supports the results from our model, reporting that the new microcirculatory network formed by regenerative angiogenesis in a skeletal muscle mouse model presented many structural and functional abnormalities even after several months. Reduced responsiveness in the operated limb is undoubtedly present due to the removal of the vessels, meaning that the non‐operated limb responds more actively to the local (also to the central reflexes) effects of hyperoxia. Nevertheless, regional perfusion dynamics seems also to take place to recover the equivalent circulatory steady state in both limbs. A similar cooperation perfusion event in both limbs has been described in human and interpreted as a long‐term vascular adaptation to ischemia in patients with peripheral arterial occlusive disease (Bongard et al. 1992), but to our knowledge, this has not been experimentally pursued. The impact of postural vasoconstriction in the human foot, measured with LDF, revealed a pronounced reduction of perfusion in the dependent foot followed by smaller reduction in the contralateral foot, likely involving local neurogenic and myogenic mechanisms acting with central components, via sympathetic efferents (Hassan and Tooke 1988). Furthermore, a very recent study suggests that massage mobilization of the hind limb also affects the perfusion on the non‐dependent limb (Rocha et al. 2017).

## Conclusion

This study contributes to renew the interest of hyperoxia as an experimental challenger in vascular medicine and to expand its applicability. Our functional approach shows that the reproducibility of the model can be improved by reducing its variability. More importantly, with the association to the HLI model, we have illustrated its discriminative capacity by revealing that the apparent structural reestablishment obtained by Day 21 in the ischemic muscle after HLI is not followed by a perfusion normalization, most likely because these new vessels seem to have not yet acquired all normal functional competences. The perfusion balance between both limbs illustrated by the mixed perfusion responses in the control group, as during HLI recovery leading to a new circulatory steady state, is a novel, intriguing result which, we believe, should be further investigated.

## Conflict of Interest

None declared.
